# Neural mechanisms underlying visual attention to health warnings on branded and plain cigarette packs

**DOI:** 10.1111/add.13699

**Published:** 2017-01-04

**Authors:** Olivia M. Maynard, Jonathan C. W. Brooks, Marcus R. Munafò, Ute Leonards

**Affiliations:** ^1^ School of Experimental PsychologyUniversity of BristolBristolUK; ^2^ MRC Integrative Epidemiology Unit (IEU)University of BristolBristolUK; ^3^ UK Centre for Tobacco and Alcohol StudiesNottinghamUK; ^4^ Clinical Research and Imaging CentreUniversity of BristolBristolUK

**Keywords:** Attention, eye‐tracking, fMRI, health warnings, plain packaging, policy, standardised packaging, smoking, tobacco, tobacco control

## Abstract

**Aims:**

To (1) test if activation in brain regions related to reward (nucleus accumbens) and emotion (amygdala) differ when branded and plain packs of cigarettes are viewed, (2) test whether these activation patterns differ by smoking status and (3) examine whether activation patterns differ as a function of visual attention to health warning labels on cigarette packs.

**Design:**

Cross‐sectional observational study combining functional magnetic resonance imaging (fMRI) with eye‐tracking. Non‐smokers, weekly smokers and daily smokers performed a memory task on branded and plain cigarette packs with pictorial health warnings presented in an event‐related design.

**Setting:**

Clinical Research and Imaging Centre, University of Bristol, UK.

**Participants:**

Non‐smokers, weekly smokers and daily smokers (*n* = 72) were tested. After exclusions, data from 19 non‐smokers, 19 weekly smokers and 20 daily smokers were analysed.

**Measurements:**

Brain activity was assessed in whole brain analyses and in pre‐specified masked analyses in the amygdala and nucleus accumbens. On‐line eye‐tracking during scanning recorded visual attention to health warnings.

**Findings:**

There was no evidence for a main effect of pack type or smoking status in either the nucleus accumbens or amygdala, and this was unchanged when taking account of visual attention to health warnings. However, there was evidence for an interaction, such that we observed increased activation in the right amygdala when viewing branded as compared with plain packs among weekly smokers (*P* = 0.003). When taking into account visual attention to health warnings, we observed higher levels of activation in the visual cortex in response to plain packaging compared with branded packaging of cigarettes (*P* = 0.020).

**Conclusions:**

Based on functional magnetic resonance imaging and eye‐tracking data, health warnings appear to be more salient on ‘plain’ cigarette packs than branded packs.

## Introduction

Legislation mandating plain (standardized) packaging of cigarettes has now been passed in Australia, Ireland, the United Kingdom and France. A number of other countries, including Norway, Hungary, Canada, South Africa and New Zealand, are also considering this legislation. Plain cigarette packaging increases visual attention towards health warning labels (HWLs) and away from branding among non‐smokers and non‐daily smokers, but not daily smokers 1, 2, who appear to actively avoid HWLs 3. Electroencephalography (EEG) data suggest that this avoidance leads to (or perhaps is a result of) reduced processing of the emotional content of HWLs 4. Other behavioural research has shown that plain packaging reduces the ability of cigarette packs to act as a cue‐eliciting stimulus 5 and decreases pack appeal 6, 7. These differences between plain and branded packs may lead to differences in neural activity.

As a result of the attenuated incentive value of the standardized branding on plain packs compared with branded packs among smokers 5, plain packs may reduce activation in brain areas related to reward, including the ventral striatum and nucleus accumbens among smokers. The ventral striatum is associated with drug craving 8 and reward processing 9, and the nucleus accumbens, situated within the ventral striatum, is sensitized by repeated exposure to addictive drugs 10. Smoking‐related cues (e.g. cigarette packs) have been shown to activate the nucleus accumbens in daily smokers, but not non‐smokers 11. Furthermore, as a result of the increased salience of the HWL on plain cigarette packs among non‐daily smokers 1, 2, plain packs may increase activation in regions of the brain related to fear and emotion processing among these individuals compared with daily smokers. The amygdala is therefore a key region of interest, as it plays a central role in the emotional processing of sensory stimuli 12, predominately fear 13, 14 and unpleasant stimuli 15, 16. Visual stimuli containing anti‐smoking information have been shown to activate the amygdala 17, and this has been linked to smoking cessation success 18. Previous functional magnetic resonance imaging (fMRI) studies indicate that pictorial HWLs activate large‐scale neural networks 19, including the amygdala 20, 21.

To the best of our knowledge, only one study has compared neural activity in response to branded and plain packaging previously, finding no differences 20. However, this study tested only daily smokers, for whom a difference between branded and plain packs is least likely to be expected, based on our previous findings 1, 2. In this fMRI study, we aimed to extend previous research by investigating neural responses to plain and branded cigarette packaging among non‐smokers, weekly smokers and daily smokers. Given differences in visual attention when viewing plain and branded packs 1, 2, 3 and the potential impact of this on processing of the HWLs 4, we used eye‐tracking to measure visual attention to the HWLs. Combining fMRI and eye‐tracking in this way allowed us to examine the impact that visual attention to the two contrasting elements of the cigarette pack (the branding and the HWL) has on fMRI signal change. Our aims were to (1) estimate whether activation in brain regions related to reward (nucleus accumbens) and emotion (amygdala) differ when branded and plain packs of cigarettes are viewed, (2) test whether these activation patterns differ by smoking status and (3) examine whether activation patterns differ as a function of visual attention to health warning labels on cigarette packs. We hypothesized that viewing plain compared with branded packs would lead to decreased nucleus accumbens activation among daily and weekly smokers, but not among non‐smokers, and that increased visual attention to HWLs would be associated with reduced nucleus accumbens activation, as increased attention to warnings must result in decreased attention to branding; a rewarding stimulus 5. We also hypothesized that viewing plain compared with branded packs would lead to increased amygdala activation among non‐smokers and weekly smokers, but not among daily smokers, and that increased visual attention to HWLs would be associated with increased amygdala activation among non‐daily smokers, given the fear‐inducing nature of these warnings 22.

## Methods

### Design and overview

In this cross‐sectional observational study, non‐smokers, weekly smokers and daily smokers performed a memory task on branded and plain cigarette packs presented in an event‐related design. Brain activity was recorded with a 3 T MRI scanner, incorporating on‐line eye‐tracking. Testing took place at the University of Bristol Clinical Research and Imaging Centre, and ethics approval was granted by the Faculty of Science Research Ethics Committee (011011688C).

### Participants

Participants (*n* = 72) were recruited from the general population and were required to report being either non‐smokers (*n* = 24; smoking fewer than 100 cigarettes in their life‐time), weekly smokers (*n* = 24; smoking at least one cigarette per week, but not daily) or daily smokers (*n* = 24; smoking at least five cigarettes per day and within 1 hour of waking). Participants were also aged between 18 and 40 years, had normal or corrected‐to‐normal vision, were right‐handed and were free of any known neurological or psychiatric conditions and any contraindications for MRI.

The sample size for the study was calculated based on eye‐tracking data obtained in a previous behavioural study 1. This showed that daily smokers attend to branding preferentially compared with health warnings on branded packs [mean difference health warning – branding = −3.8 fixations, standard deviation (SD) = 10.8], while non‐smokers do not (mean difference = 2.8 fixations, SD = 5.4). Given that behavioural measures are likely to be less sensitive than physiological measures such as fMRI, we designed this study to be able to detect an effect of at least the magnitude observed in the behavioural study (dz = 0.7) at an alpha level of 5% and with 80% power, such that 18 participants were required in each group. We recruited 25 per group to allow for participant dropouts and exclusions (e.g. due to failure to eye‐track).

### Materials

**Figure 1:**
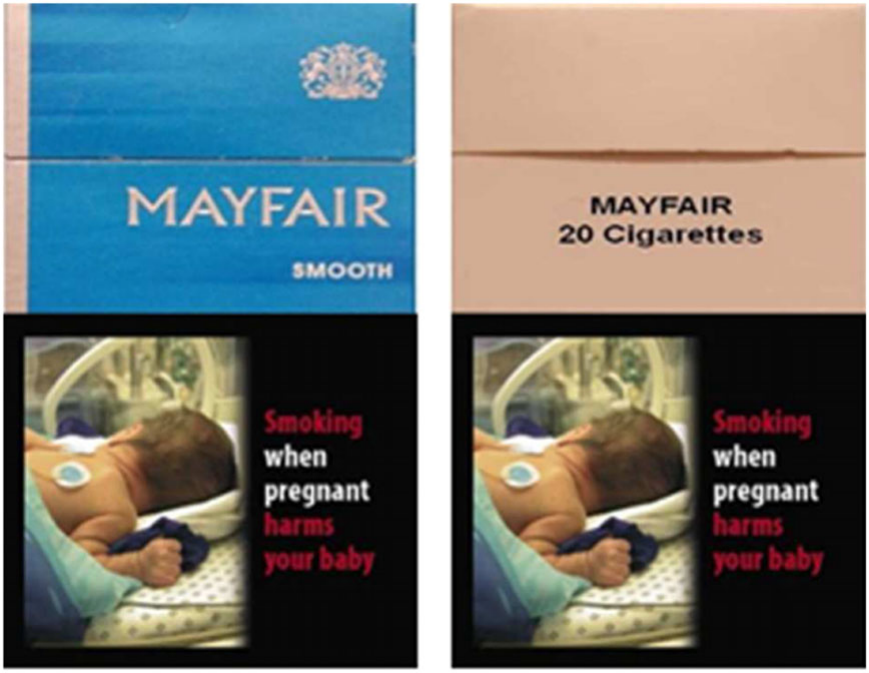
Examples of branded and plain stimuli. [Colour figure can be viewed at wileyonlinelibrary.com]

Visual stimuli of branded and plain packs (see Fig. 1) were identical to those used previously 1, 2. Each of 10 different cigarette pack brand labels (in both ‘branded’ and ‘plain’ formats) were combined with 32 HWL images (creating a total of 640 stimuli), taken from the European Commission set of 35 pictorial HWLs. The most effective HWLs were selected, based on pre‐study piloting 3. Control stimuli were a single phase‐scrambled image of a cigarette pack with a red fixation cross in the centre, and a blank screen with the same fixation cross.

### Procedure

Following informed consent, smoking status was verified using a carbon monoxide (CO) breath test. Smokers completed the Questionnaire of Smoking Urges–Brief (QSU) 23 and the Fagerström Test for Nicotine Dependence (FTND) 24. Image data were acquired with a Siemens Skyra 3 T MR system with a 32‐channel receive‐only head coil, and participants' eye movements were recorded using an Eyelink 1000 eye‐tracker (SR Research Ltd, Ottawa, Ontario, Canada) at a sampling rate of 1000 Hz and typical spatial resolution of < 0.4 degrees of visual angle. During scanning, cigarette packs were presented individually in an event‐related design. In order to maintain participant engagement, participants completed a visual working‐memory task on these cigarette pack images 1, 2. Over eight blocks, participants viewed 128 cigarette pack stimuli (16 per block: eight branded and eight plain packs intermixed randomly). Stimuli were selected pseudo‐randomly from the larger set of 640 stimuli, such that each HWL was presented four times over the experiment, twice with each of the branded and plain packs, for 4 seconds each. Control stimuli were presented after each cigarette pack (either the phase‐scrambled cigarette pack or the fixation cross, presented pseudo‐randomly such that each was presented with equal frequency per block) for durations ranging randomly between 4 and 10 seconds. To ensure their engagement, participants were told that their memory for the test stimuli would be assessed during a recall phase after each block. Prior to the recall phase, instructions were presented for 5 seconds. During the recall phase, two cigarette pack stimuli (either branded or plain) were presented consecutively on screen, and using a hand‐held button‐box participants were given up to 5 seconds to respond whether they had seen these images in the preceding block. After each recall phase, instructions informed participants that the next block was about to commence. Before the fifth block, participants were given a 20‐second rest. Following the scan, participants were asked to provide demographic information, and smokers completed the QSU. All participants were then debriefed fully, encouraged to ask questions and reimbursed with £20.

### Image acquisition

During functional imaging, echo planar T2* weighted images (EPIs) were acquired in a transversal direction, prescribed parallel to the anterior commissure–posterior commissure (AC–PC) line [50 slices, repetition time (TR) = 3000 ms, echo time (TE) = 30 ms, flip angle = 90°, field of view (FOV) = 192 mm^2^, imaging matrix = 64^2^, slice thickness = 3 mm, slice gap = 0 mm, voxel size = 3 mm^3^]. After the main experimental task, a three‐dimensional T1 weighted Magnetization‐Prepared Rapid Gradient Echo (MPRAGE) image volume was acquired (192 slices, TR = 1800 ms, TI = 2200 ms, TE = 2.25 ms, imaging matrix = 256 × 256, FOV = 240 mm^2^, flip angle = 9°, slice thickness = 0.9 mm and voxel size = 0.9 mm^3^), which was used to aid registration to the 2‐mm resolution Montreal Neurological Institute‐152 (MNI‐152) standard brain template. For the purpose of EPI distortion correction, a fieldmap was acquired using a gradient‐recalled dual echo sequence (49 transversal slices, TR = 520 ms, TE1/TE2 = 4.92/7.38 ms, slice thickness = 3 mm, flip angle = 20°, FOV = 192 mm^2^ **,** voxel size = 3 mm^3^).

### MRI pre‐processing and data analysis

Pre‐processing and analysis of fMRI data was performed using FSL version 5.0.4 25 and FMRIB's Expert Analysis Tool 6.00 (FEAT). Pre‐processing included motion correction using FMRIB's Linear Image Registration Tool (McFLIRT), spatial smoothing with a 5‐mm full‐width half‐maximum Gaussian kernel, high‐pass temporal filtering with a 110‐second cut‐off and spatial distortion correction of functional images using fieldmaps. Responses to stimulation were estimated using the general linear model in FEAT, accounting for motion outliers 26.

**Figure 2:**
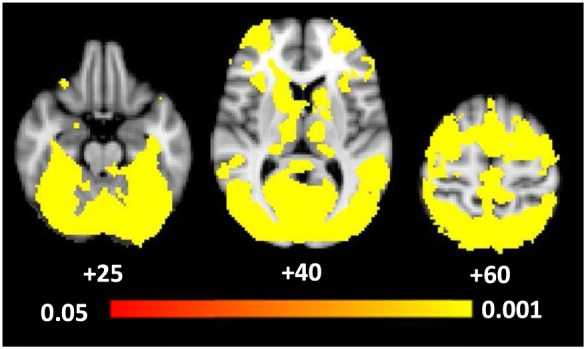
Blood oxygen level‐dependent (BOLD) activation for the confirmatory analysis examining the effect of the task (i.e. branded and plain packs versus control stimuli). Coordinates represent *Z* coordinates in Montreal Neurological Institute‐152 (MNI‐152) coordinate space. The colour bar represents significance levels with the intensity representing the *P*‐values

During first‐level analysis, each event in the trial sequence was modelled with separate explanatory variables (EVs) (i.e*.* all instructions, phase‐scrambled images, recall images, button‐box responses, branded and plain package images). Note that we did not include contrasts with phase‐scrambled stimuli to control for visual differences between branded and plain images in our primary analysis, as we were interested to see whether visual elements of the cigarette packs themselves would counteract attentional effects to HWLs differently for the smoking groups. Separately for the branded and plain package stimuli, two EVs were created: one where each individual stimulus was weighted equally (i.e. ‘1’, hereafter ‘equally weighted’) and one where each was weighted according to the percentage of fixations on the HWL compared with the branding (hereafter ‘eye‐tracking weighted’), giving a total of four cigarette package EVs. Only participants for whom sufficiently high quality eye‐tracking data were obtained during experiments were included in the analyses. Individual trials were excluded (using first‐level analysis confound EVs) if the connection to the eye‐tracker was lost at any point during the trial, or if the participant had had their eyes closed for more than half the trial. We initially performed an analysis to confirm that our task (i.e. exploring and memorizing cigarette packs) activated attention and working memory networks. For this, first‐level contrasts combining activation to branded and plain packs were calculated and compared to activation for control stimuli. These were combined at higher‐level analysis to calculate the mean activation for the contrast cigarette packs versus control (cluster‐forming threshold *Z* = 0.39). Our control stimuli consisted of images with phase‐scrambled cigarette packages to ensure that from a sensory perspective there was no difference between control images and cigarette packs (note, however, that the perceptual input between cigarette packages and phase‐scrambled packages differs). The pattern of activation for this high‐level analysis is shown in Fig. 2; the key structures activated were the frontal pole, pre‐central gyrus and lateral occipital cortex (superior and inferior division), indicating clear visual attention‐related network activation, as expected for a task that requires image exploration and working memory processes (e.g. 27, 28).

For our primary analysis, first‐level analyses yielded images of beta weights (‘contrast images') for the blood oxygen level‐dependent (BOLD) response to branded and plain packaging. The beta‐weights obtained from the equally weighted EVs reflect the mean response to each stimulus type, controlling for the number of fixations to the health warnings, which is achieved by including eye‐tracking weighted EVs into the first‐level model. The beta weights obtained from the eye‐tracking weighted EVs allowed us to examine the impact of increased visual attention to the HWLs on neural activation, and relate to the slope (i.e. the sign and magnitude) of the relationship between BOLD and visual attention (i.e. this regressor accounted for variation in the BOLD response that depended on the amount of time spent fixating to HWLs). Analyses are split into those conducted using the equally weighted and eye‐tracking weighted models.

To facilitate group analysis, contrast images were normalized to the MNI‐152 anatomical standard. Briefly, the Brain Extraction Tool (BET) was used to remove non‐brain tissue from each subject's high‐resolution T1 weighted structural image, and the undistorted (i.e. fieldmap corrected) functional images co‐registered to the brain extracted structural scan using boundary‐based registration (BBR) 29. Participants' structural scans were normalized to the anatomical standard using both affine (FLIRT) and non‐affine transformations (FMRIB's Non‐linear Image Registration Tool, FNIRT).

Anatomically masked and whole‐brain voxel‐wise group analyses were performed using Permutation Analysis of Linear Models (PALM) software 30, 31 to determine family‐wise error (FWE)‐corrected significance levels. Pre‐specified brain masks (bilaterally in the amygdala and nucleus accumbens) were defined on the basis of anatomically specified regions from the probabilistic Harvard–Oxford Subcortical Structural Atlas thresholded at 30%. Within PALM, the following options were used: exchangeability blocks 32, within‐ and whole‐block permutation, 500 permutations (with the command ‘‐approx tail’ for faster inference) 33, threshold‐free cluster enhancement (TFCE) 34 and FWE correction of *P*‐values over multiple contrasts, taking into account any dependency that may exist between these contrasts (using the command ‘‐corrcon’) 34. This command meant that testing the *F*‐test explicitly for the interactions and main effects was unnecessary, and instead a series of corrected contrasts assessed the nature of these. Contrasts examined differences by pack type (i.e. branded > plain and plain > branded), smoking status (i.e. each of the three groups compared with the two others) and the interaction between these (i.e. branded > plain and plain > branded for each smoking group and the differences between the three smoking groups for both branded and plain packs). Two separate group analyses using contrast images obtained from both the equally weighted and the eye‐tracking weighted EVs were run. All reported results are based on the corrected TFCE output from PALM.

## Results

### Characteristics of participants

A total of 72 participants were tested (see Table 1 for characteristics). The data of 14 participants (five non‐smokers, five weekly smokers and four daily smokers) were removed from all analyses, as more than half of their trials were excluded based on the eye‐tracking data exclusion criteria (these exclusions were due largely to the participants closing their eyes). Data from 19 non‐smokers, 19 weekly smokers and 20 daily smokers were therefore available for analysis. For the remaining participants, individual trials violating one of the exclusion rules were removed from all further analyses. Of the 128 trials per participant, the average number of excluded trials was 13 (SD = 1.6). The groups were similar on demographic variables (age and sex) and percentage of correct responses in the recall phase, and differed as expected on measures of smoking heaviness, dependence and craving, except for intention to quit, where they were similar (see Table 1).

**Table 1:** Characteristics of participants.

	Non‐smokers (*n = 19)*	Weekly smokers (*n = 19)*	Daily smokers (*n = 20)*	*P‐value for difference*
Sex (male)	10 (53%)	10 (53%)	11 (55%)	0.99
Age	24∙0 (3∙0)	21∙3 (2∙8)	22∙6 (4∙2)	0∙06
Exhaled carbon monoxide	2∙2 (1∙2)	4∙1 (3∙0)	13∙0 (6∙6)	< 0∙001
Quitting contemplation ladder	NA	4∙8 (1∙8)	4∙6 (1∙2)	0∙55
Fagerström Test for Nicotine Dependence (FTND)	NA	0∙1 (0∙2)	2∙1 (1∙7)	< 0∙001
Questionnaire of smoking urges (QSU)—Brief (pre‐scan)	NA	24∙0 (8∙2)	32∙5 (12∙7)	< 0∙001
Questionnaire of smoking urges (QSU)—Brief (post‐scan)	NA	24∙1 (12∙6)	40∙8 (13∙0)	< 0∙001
Percentage correct recall	76∙0 (10∙4)	76∙6 (13∙3)	70∙9 (13∙3)	0∙06
Cigarettes smoked	NA	10∙9/week (6∙7)	81∙5/week (36∙0)	< 0∙001
			11∙7/day (5∙1)	

Values represent number (percentage) for categorical variables, and mean (standard deviation) for continuous variables. *P*‐values for the difference between groups was calculated using χ^2^, *F*‐ or *t*‐tests, as appropriate. NA = not applicable.

### Eye‐tracking analyses

**Figure 3:**
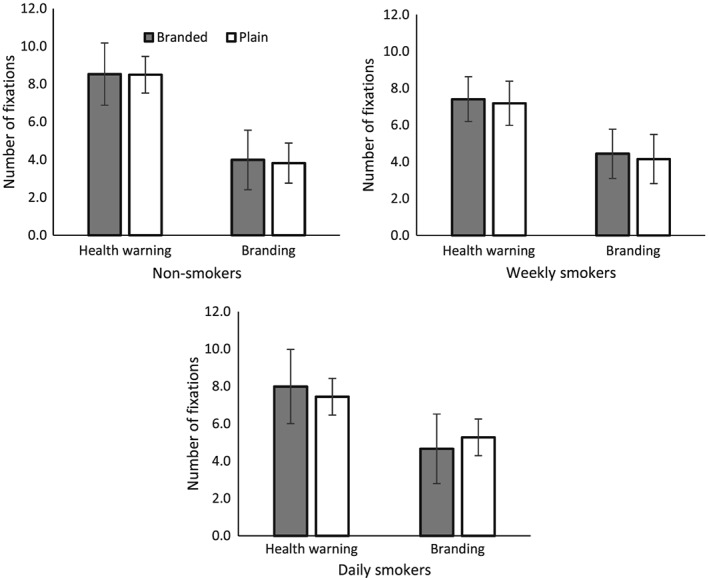
Mean number of fixations on branding and health warnings on branded and plain packs among non‐smokers, weekly smokers and daily smokers. Error bars represent adjusted 95% confidence intervals

A 3 (smoking status: non‐smoker, weekly smoker, daily smoker) × 2 (location of fixation: branding, HWL) × 2 (pack type: plain packs, branded packs) analysis of variance (ANOVA) on number of fixations (see Fig. 3) indicated a main effect of location (*F*_(1,55)_ = 177.65, *P* < 0.001, η^*2*^ *P* = 0.76), reflecting more fixations on the HWL [mean = 7.8, standard error (SE) = 0.2] versus branding (mean = 4.4, SE = 0.1). A main effect of pack type was also observed (*F*_(1,55)_ = 7.65, *P* = 0.01, η^*2*^ *P* = 0.12), although the absolute difference between branded (mean = 6.2, SE = 0.1) and plain packs (mean = 6.1, SE = 0.1) was small. No clear evidence for a main effect of smoking status (*F*_(1,55)_ = 1.86, *P* = 0.17, η^*2*^ *P* = 0.06) was observed.

A pack type × location × smoking status interaction was observed [*F*_(2,55)_ = 7.18, mean squared error (MSE) = 0.36, *P* = 0.002, η^*2*^ *P* = 0.21]. Further analyses, stratified by smoking status, indicated a pack type × location interaction for daily smokers (*F*_(1,19)_ = 25.97, MSE = 0.26, *P* < 0.01, η^*2*^ *P* = 0.58), but not weekly smokers (*F*_**(**1,18)_ = 0.04, MSE = 0.58, *P* = 0.85, η^*2*^ *P* = 0.002) or non‐smokers (*F*_(1,18)_ = 0.33, MSE = 0.25, *P* = 0.57, η^*2*^ *P* = 0.02), Bonferroni‐corrected *post‐hoc* tests indicated that, for daily smokers, this interaction was characterized by more fixations on the branding on plain packs versus branded packs (*t*_(19)_ = 4.62, *P* < 0.001).

### Anatomically masked analyses

#### Equally weighted analyses

**Figure 4:**
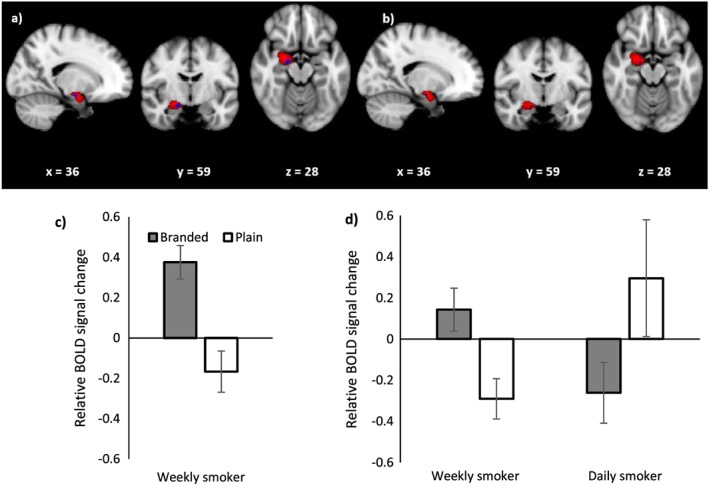
(a) Blood oxygen level‐dependent (BOLD) activation in the right amygdala for the comparison plain > branded controlling for fixations to health warnings (i.e. eye‐tracking weighted analysis) among weekly smokers and (b) weekly smokers compared with daily smokers. Coordinates represent coordinates in Montreal Neurological Institute‐152 (MNI‐152) coordinate space. The red region shows the extent of the 30% threshold applied to the probabilistic right amygdala mask and the blue region is the activated cluster. (c) Relative BOLD signal change in the activated region of the right amygdala for the equally weighted analysis for the comparison branded > plain for weekly smokers. (d) Relative BOLD signal change in the activated region of the right amygdala for the equally weighted analysis for the comparison weekly smokers > daily smokers. Error bars represent the standard error of the mean

Across the four regions analysed in the masked analyses, there was no evidence of a main effect of pack type (*P*s > 0.17) or smoking group (*P*s > 0.98). There was some evidence for a smoking status × pack type interaction in the right amygdala, characterized by a branded > plain difference for weekly smokers (*P* = 0.003; see Fig. 4a) but not non‐smokers or daily smokers, such that this region was activated when viewing branded packs, but deactivated when viewing plain packs (see Fig. 4c). However, the area of activity was small (the 30% thresholded amygdala mask comprised 434 voxels, while the activated region was characterized by a single cluster of nine voxels). We observed a smoking status × pack type interaction in a different subregion of the right amygdala, which was characterized by greater activity for the branded > plain contrast among weekly compared with daily smokers (*P* = 0.004; see Fig. 4b). Again, the areas of activation were small, represented by three clusters of three or fewer voxels. Figure 4d shows that the difference between weekly and daily smokers is characterized by activation within the right amygdala among weekly smokers when viewing branded packs alongside a relative deactivation when viewing plain packs, but the opposite pattern of results for daily smokers.

#### Eye‐tracking weighted analysis

Across the four regions analysed in the masked analyses there was no evidence of a main effect of pack type (*P*s > 0.90), smoking group (*P*s > 0.71) or a smoking status × pack type interaction.

### Whole brain analyses

#### Equally weighted analyses

**Figure 5:**
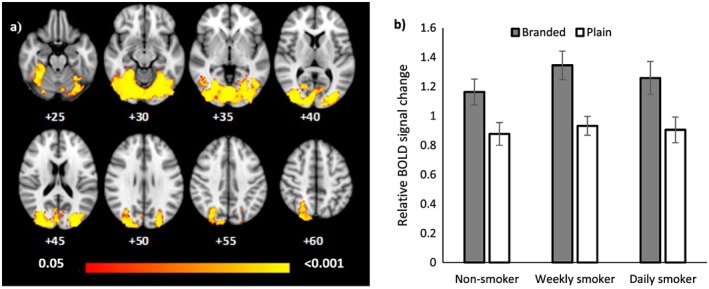
(a) Blood oxygen level‐dependent (BOLD) activation for the equally weighted analysis for the comparison branded > plain. Differences between conditions were assessed using permutation testing, and the levels of evidence presented using family‐wise error (FWE)‐corrected *P*‐values. Coordinates represent *Z* coordinates in Montreal Neurological Institute‐152 (MNI‐152) coordinate space. The colour bar represents significance levels with the intensity representing the *P*‐values. (b) Group average BOLD percentage signal difference extracted for plain and branded packaging types from the activated cluster (see Fig. 5a) for non‐smokers, weekly smokers and daily smokers. Error bars represent the standard error of the mean

The whole‐brain group analysis of the data obtained with the equally weighted EV revealed no difference for the plain > branded contrast, but a difference for the branded > plain contrast (*P* < 0.001) in the visual cortices. Evidence of this difference was observed among all three smoking groups (see Fig. 5b: non‐smoker, *P* = 0.06; weekly smoker, *P* < 0.001; daily smoker, *P* = 0.02). Using the Harvard–Oxford Cortical and Subcortical Structural Atlases and an automatic atlas query tool ‘autoaq’ 35, one cluster of activation (15 807 voxels in standard space, local maxima: *x* = 26, *y* = −78, *z* = −12) characterized this difference (Fig. 5a). The structures to which the cluster belonged (and their mean percentage probability) were identified as the lateral occipital cortex superior division (10.1%), lateral occipital cortex inferior division (8.5%), occipital pole (8.2%), lingual gyrus (7.9%) and occipital fusiform gyrus (7.7%), confirming increased activity in visual areas to branded compared to plain stimuli in line with their increased visual complexity. Only activated regions with a percentage probability of belonging to that structure of more than 5% are reported. There was no evidence of a pack type × smoking status interaction or of a main effect of smoking status (*P*s = 0.99).

#### Eye‐tracking weighted analyses

**Figure 6:**
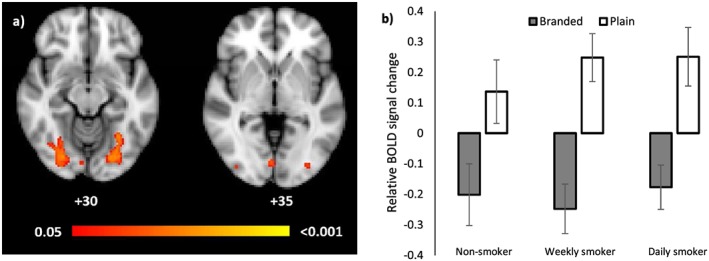
(a) Difference in relationship between blood oxygen level‐dependent (BOLD) and visual attention depending on packaging type. The image represents the comparison plain > branded for the eye‐tracking weighted analysis. The intensity levels (and colour bar) represent inference levels determined with permutation testing (see Fig. 5 legend for details). Coordinates represent *Z* coordinates in Montreal Neurological Institute‐152 (MNI‐152) coordinate space. (b) Group average beta weights (i.e. for the individual eye‐tracking weighted contrasts for plain and branded packaging) from the activated cluster (see Fig. 6a) for non‐smokers, weekly smokers and daily smokers. Error bars represent the standard error of the mean

The higher‐level whole brain analysis of the eye‐tracking weighted model revealed a main effect of packaging type for the contrast plain > branded (*P* = 0.02), but no difference for the contrast branded > plain. Three clusters of activation characterized this difference (Fig. 6a). The structures to which cluster 1 (348 voxels in standard space, local maxima: *x* = 28, *y* = −78, *z* = −12) belonged were identified as the occipital fusiform gyrus (44.4%) and lateral occipital cortex, inferior division (8.0%). The structures to which cluster 2 (332 voxels, *x* = −26, *y* = −80, *z* = −12) belonged were the occipital fusiform gyrus (38.9%), temporal occipital fusiform cortex (9.1%), lateral occipital cortex, inferior division (6.2%) and lingual gyrus (5.4%). The structures to which cluster 3 (151 voxels, *x* = 4, *y* = −82, *z* = −6) belonged were the lingual gyrus (51.3%), calcarine sulcus (12.3%) and occipital pole (6.4%). Figure 6b shows the relative BOLD signal change in these activated regions: the difference is characterized by a positive relationship between BOLD and visual attention to HWLs within these regions when viewing plain packs and a negative relationship when viewing branded packs. There was no evidence for a smoking status × pack type interaction (non‐smoker, *P* = 0.89; weekly smoker, *P* = 0.21; daily smoker, *P* = 0.86), and no main effect of smoking status (*P* = 0.99).

## Discussion

Based on our previous eye movement research 1, 2, 3, we hypothesized that viewing plain compared with branded packs would lead to decreased activation in the nucleus accumbens among smokers compared with non‐smokers, and that viewing plain compared with branded packs would lead to increased activation in the amygdala among non‐smokers and weekly smokers, but not daily smokers. By combining fMRI with eye‐tracking we were able to both control for the impact of visual attention on neural responses in the equally weighted analysis and examine the relationship between visual attention to HWLs and fMRI signal change for branded and plain packs in the eye‐tracking analysis. We found no evidence for pack‐type‐dependent activation in the nucleus accumbens and no difference between groups. This might be due to the reported technical difficulties with imaging this region, given its size and location 36. We found evidence for a smoking status × pack type interaction within the right amygdala, which was driven by opposing responses to pack types among weekly and daily smokers. While HWLs presented on plain packaging decreased activity within the right amygdala for weekly smokers, they provoked an increase among daily smokers when controlling for visual attention to warnings. This pattern of activation in the amygdala for weekly smokers was in the opposite direction to our hypothesis: we expected increased activity for plain as opposed to branded packages, reflecting increased negative emotions induced by the HWLs on plain packages. In addition, contrary to our expectations, we did not find evidence that variation in BOLD activity in the amygdala was dependent upon fixations to the HWLs (i.e. the eye‐tracking weighted analysis). The amygdala has been implicated in processing positive reward and reinforcement 37, 38 and cue–reactivity 36, in addition to processing negative stimuli. Our observations may therefore reflect a positive emotional response or cue–reactivity to the branded compared with plain packs among weekly smokers. However, why we observed the opposite response among daily smokers is not clear. Furthermore, as we are measuring relative changes without a clear baseline, any interpretation of these reverse patterns needs to be considered with caution.

As an exploratory analysis, we also tested whether we saw increased activation in early visual areas for branded compared to plain packs. Indeed, the upper, ‘branded’ region of branded packs is more visually complex than for plain packs, and should result in increased activation in retinotopic regions 39 of the upper visual field in striate and extrastriate visual cortex compared with plain packs 36. In line with these expectations, when modelling activity with an equally weighted regressor and controlling for visual attention to health warnings, presentation of branded compared with plain packs increased activation in the visual cortex. Specifically, areas of increased activation included the lateral occipital cortex superior division, the lingual gyrus and the occipital fusiform gyrus, regions of the brain involved in image processing 40, 41, 42, 43, 44, 45 and attention‐related visual tasks 46. Our findings are consistent with a meta‐analysis of fMRI studies, which has shown that presentation of smoking‐related cues compared with neutral cues activates extended areas of the visual cortex 36, and also with research which shows that plain packs are poor cue‐eliciting stimuli 5. Further research should investigate the extent to which plain packs activate regions involved in smoking cue–reactivity 36. When modelling activity with a regressor whose weights reflected the amount of time spent looking at the HWL (i.e. the eye‐tracking weighted analysis), a different pattern of results was observed. Here a positive relationship between BOLD activity and number of fixations towards the HWL was observed for plain packs, whereas the BOLD/HWL relationship was negative when viewed on branded packaging. That increased fixations to the same stimulus (the HWL) had opposite effects on BOLD activation in the visual cortex when presented in the context of plain (activation) compared with branded packaging (deactivation) suggests that HWLs on plain packs may be more visually salient than those on branded packs. Indeed, we observed activation for this contrast in the calcarine sulcus, which is located in the primary visual cortex (V1), and is a region of the brain important in visual attention 47. This interpretation supports and extends our previous findings that plain packaging increases visual attention to HWLs 1, 2, 3, and indicates that this increase may be as a result of the visual salience of the HWLs on plain packs.

There are a number of limitations of our study. First, as 14 participants were removed from the analysis due to unavailability of eye‐tracking data, the sample size was smaller than intended originally. Given that the combination of eye‐tracking with fMRI was a key strength of this study, it was important to obtain quality data from all participants. Moreover, our final sample size of 58 participants is still relatively large for an imaging study in this field. However, this sample size did not permit comparisons across other groups (i.e. age or gender). Secondly, we did not observe any differences in activation in the nucleus accumbens between our smoking groups or across pack types. Had we shown smokers their own cigarette brand, arguably a more rewarding stimulus than all brands aggregated together, we might have observed differences in this region. Participants' own brands were not used due to the expected effects of habituation after repeated exposure. Future experiments may consider using more ecologically valid stimuli, perhaps in naturalistic scenes, to overcome this limitation. It is also important to note that the daily smokers in this study reported low levels of nicotine dependence. As a result, the cigarette packs may not have been particularly rewarding stimuli for these participants. In addition, we did not standardize time since last cigarette for smokers in this study. Although the mean CO reading for daily smokers suggested that smokers had smoked a cigarette relatively recently prior to the start of the study, we are not able to determine which participants might have been experiencing nicotine withdrawal and, as a result, for which participants the cigarette packages may have been particularly rewarding. It is also important to note that although CO level may have influenced BOLD activity, the evidence for this is currently unclear 48, 49. Thirdly, we did not record participants' self‐reported responses to the stimuli, which may have provided additional context for the results, or measures of other substance use, which may have influenced baseline blood flow in reward‐related brain regions. Furthermore, we can only speculate that activation in the visual cortex for the plain > branded comparison for the eye‐tracking weighted analysis is related to visual processing of the HWL. An analysis using retinotopic mapping 50 may have helped to identify the specific regions within the visual cortex which were related to attention to the branding and the HWL.

Ours is the first study to integrate fMRI with eye‐tracking to investigate the effects of plain versus branded cigarette packaging on neural activity while controlling for visual attention to HWLs. Research which uses these objective methods of assessing the probable effects of plain packaging are less likely to be influenced by the biases potentially present in some other research in this field relying upon subjective responses 6, 51, and may therefore be important in informing public policy. In our relatively large sample of non‐smokers, weekly smokers and daily smokers, we find that when taking into account visual attention to HWLs, plain packaging compared with branded packaging leads to increased activation in a large neural network in the visual cortex. We suggest that plain packaging might increase the visual salience and visual processing of HWLs on cigarette packs. This finding extends previous research, which shows that plain packaging might be an effective tobacco control strategy 6, 51, and strengthens the case for plain packaging in countries considering this legislation world‐wide.

## Declaration of interests

None.
